# Role of Post-translational Modifications in Influenza A Virus Life Cycle and Host Innate Immune Response

**DOI:** 10.3389/fmicb.2020.517461

**Published:** 2020-09-04

**Authors:** Jiao Hu, Lei Zhang, Xiufan Liu

**Affiliations:** ^1^Animal Infectious Disease Laboratory, School of Veterinary Medicine, Yangzhou University, Yangzhou, China; ^2^Jiangsu Co-innovation Center for Prevention and Control of Important Animal Infectious Diseases and Zoonosis, Yangzhou University, Yangzhou, China; ^3^Key Laboratory of Prevention and Control of Biological Hazard Factors (Animal Origin) for Agrifood Safety and Quality, Ministry of Agriculture of China, Yangzhou University, Yangzhou, China

**Keywords:** influenza virus, post-translational modifications, replication cycle, pathogenesis, virus–host interaction, innate immune response

## Abstract

Throughout various stages of its life cycle, influenza A virus relies heavily on host cellular machinery, including the post-translational modifications (PTMs) system. During infection, influenza virus interacts extensively with the cellular PTMs system to aid in its successful infection and dissemination. The complex interplay between viruses and the PTMs system induces global changes in PTMs of the host proteome as well as modifications of specific host or viral proteins. The most common PTMs include phosphorylation, ubiquitination, SUMOylation, acetylation, methylation, NEDDylation, and glycosylation. Many PTMs directly support influenza virus infection, whereas others contribute to modulating antiviral responses. In this review, we describe current knowledge regarding the role of PTMs in different stages of the influenza virus replication cycle. We also discuss the concerted role of PTMs in antagonizing host antiviral responses, with an emphasis on their impact on viral pathogenicity and host range.

## Introduction

**FIGURE 1:**
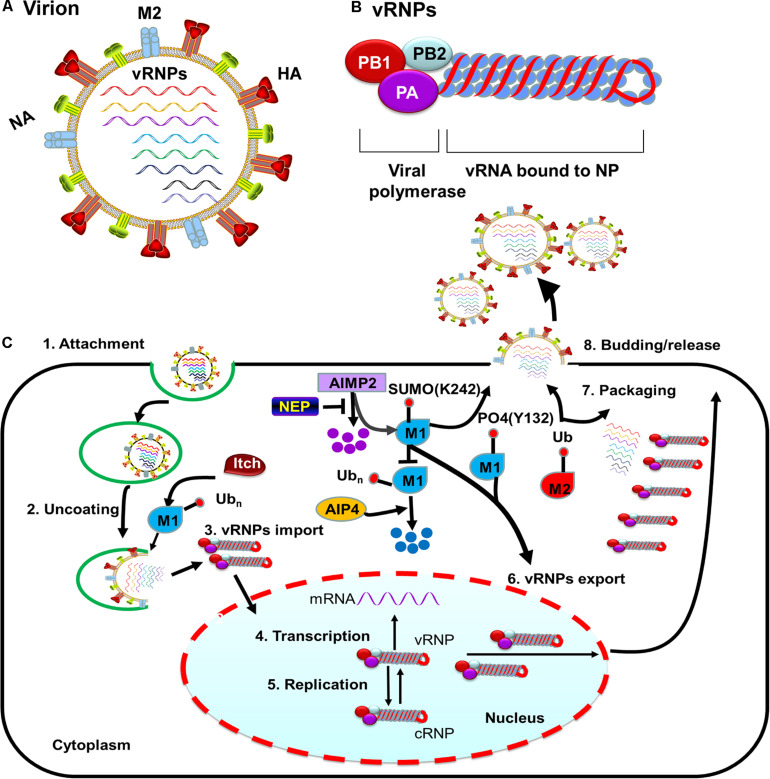
M protein-associated PTMs that regulate the influenza virus life cycle. **(A)** Diagram of influenza A viruses (IAV). IAV are enveloped viruses containing eight single-stranded negative-sense RNA gene segments that each encodes one or more proteins. The viral membrane proteins HA, NA, and M2 are shown, along with eight viral ribonucleoproteins (vRNPs). **(B)** The structure of influenza virus vRNPs. The vRNPs of influenza virus are composed of genomic RNA (red line) bound at both ends by the trimeric viral polymerase (containing PB1, PB2, and PA) and coated by oligomers of NP (blue ball). **(C)** PTMs associated with M1 or M2 that regulate the influenza virus life cycle. Ubiquitination of M1 by the E3 ubiquitin ligase Itch promotes virus uncoating, thus facilitating vRNPs release from endosomes. M1 SUMOylation at K242 and phosphorylation at Y132 promote vRNPs export. The host protein AIMP2 changes the modification state of M1 from ubiquitinated to SUMOylated. NEP alleviates the ubiquitination of AIMP2; leading to enhanced M1 import and subsequent efficient vRNPs export. M2 ubiquitination facilitates the packaging of the viral genome. M1 SUMOylation at K242 and M2 ubiquitination at K78 contribute to viral assembly and maturation. Abbreviations: SUMO, SUMOylation; Ub, ubiquitin and ubiquitination; PO4, phosphorylation; vRNP(s), viral ribonucleoprotein(s); mRNA, messenger RNA; AIMP2, aminoacyl tRNA synthase complex-interacting multifunctional protein 2; AIP4, atrophin-1-interacting protein 4; HA, hemagglutinin; M1, viral matrix 1; M2, viral matrix 2; NA, neuraminidase; NEP, nuclear export protein.

Influenza A viruses (IAV) are enveloped viruses containing eight single-stranded, negative-sense RNA gene segments. Each viral gene encodes one or more proteins. The viral surface is decorated with the envelope proteins, i.e., HA, NA, and matrix 2 (M2). The matrix 1 (M1) provides support underneath the envelope. Individual viral ribonucleoproteins (vRNPs) complexes containing viral RNA (vRNA) from the eight gene segments reside inside each virion (Figure 1A). The vRNPs are composed of genomic RNA bound at both ends by the trimeric viral polymerase containing PB1, PB2, and PA and coated by oligomers of NP (Figure 1B). Drug-resistant influenza viruses commonly arise as a result of frequent genetic changes, which can lead to antiviral drug resistance. Therefore, development of new effective antiviral therapies against influenza viruses is urgently needed. Owing to its relatively small genomic-coding capacity and the nature as an obligate intracellular parasite, IAV relies heavily on host cellular functions and must coopt host cellular machineries to support all stages of its life cycle, from entry to viral budding out of the infected cells. Additionally, influenza viruses either counteract or exploit different complex cellular mechanisms to establish an efficient infection and achieve survival.

Post-translational modifications (PTMs) substantially affect protein function through a wide variety of regulatory mechanisms, such as modulating protein shape, regulating cellular localization, and affecting protein interactions with other macromolecules. The functions of influenza virus proteins are finely regulated by diverse types of PTMs, most notably phosphorylation, ubiquitination, SUMOylation, and acetylation (Fu et al., 2015; Rudnicka and Yamauchi, 2016; Giese et al., 2017; Zheng et al., 2017; Dawson and Mehle, 2018; Tan and Gao, 2018). Phosphorylation is a common mechanism by which pathway cascades are modified, and it is used to control many key features, such as multiprotein assembly formation, protein stability, and enzymatic activity (Deribe et al., 2010). Ubiquitination is a reversible PTM that depends on the concerted action of ubiquitinating and deubiquitinating enzymes (Calistri et al., 2014). The modification process of SUMOylation is similar to that of ubiquitination; modifications resulting from both these processes regulate protein interactions by modifying the substrate proteins. In addition, SUMO can competitively bind the substrate proteins of ubiquitin. Both ubiquitination and SUMOylation are widely involved in various cellular processes, including DNA damage repair, transcriptional regulation, protein function regulation, and protein–protein interaction, as well as in various signaling pathways, such as those linked to gene regulation, epigenetics, cell differentiation, protein degradation, and tumorigenesis; they also play roles in viral replication and immune responses (Johnson, 2004; Pickart, 2004; Welchman et al., 2005). Acetylation is another major class of PTMs that is carried out by lysine acetyltransferases and reversed by lysine deacetylases to control various cellular and viral protein functions (Marazzi et al., 2012; Drazic et al., 2016). Other PTMs, such as glycosylation (de Vries et al., 2010; Herfst et al., 2012), NEDDylation (Hughes et al., 2015; Zhang et al., 2017), and ISGylation (Tang et al., 2010; Zhao et al., 2010) also play important roles in regulating protein function and contribute to processes involved in viral infection.

During IAV infection, host cells can accelerate the immune response by changing the functions of existing proteins via PTMs. However, viruses also use PTMs to break through these defenses, replicate efficiently, and disseminate quickly to new host cells. Many PTMs have been identified as central players in mediating the host innate immune response to influenza virus infection (Rajsbaum et al., 2012; Xing et al., 2016; Koliopoulos et al., 2018; Winkler et al., 2019). Such events are often beneficial for virus replication and/or constitute immune evasion strategies associated with efficient viral gene expression and virus dissemination in the host. Understanding how influenza virus uses PTMs system for efficient infection may identify novel targets for pharmacological intervention. Here, we discuss how IAV uses PTMs to accomplish every step of the virus replication cycle, from viral entry into the host cell, replication and transcription of the genome, nucleocytoplasmic shuttling of ribonucleoprotein components, transport of newly synthesized proteins, assembly of new particles, to budding and release from the infected cell. We also discuss how the IAV usurps the PTMs system to evade host innate immune responses, thus accelerating viral pathogenesis.

## PTMs Involved in the Viral Entry Process of IAV

To successfully enter the host cell, influenza virus goes through a multistep entry process (Edinger et al., 2014). This process has six fundamental steps: (1) The virus attaches to the cell through the binding of the envelope protein HA to the sialic acid receptor on the cell surface; (2) Upon attachment, the virus is internalized into the cell, mainly through Clathrin-mediated endocytosis (Matlin et al., 1981; Yoshimura et al., 1982); (3) The virus is transported from early endosomes to late endosomes through endosomal trafficking (Rust et al., 2004; Cocucci et al., 2012); (4) Viral and endosomal membranes fuse in a low-pH environment through a process that is HA-dependent and is essential for releasing vRNPs into the cytoplasm (Maeda and Ohnishi, 1980; Daniels et al., 1985; White and Wilson, 1987); (5) The vRNPs are released from the structured layer of M1 and then enter into the cytoplasm through a process called viral uncoating; (6) Last, vRNPs are transported into the nucleus, marking the end of the viral entry process and the beginning of the nuclear phase of the replication cycle. Most of these influenza virus entry steps depend critically on the host cells, and numerous PTMs have been identified in recent years as being instrumental during this process (Table 1).

**TABLE 1:** Roles of post-translational modifications during the influenza A virus life cycle.

**Stage of virus life cycle**	**Host factors^*a*^**	**PTMs**	**Biological effect**	**Reference^*b*^**
Entry/binding	/	Glycosylation of HA	Affects affinity for sialic acid receptor	de Vries et al., 2010
Entry/internalization	Akt	Phosphorylation of NS1 at T215	Increases viral internalized efficiency and viral replication	Hirata et al., 2014
	Epsin 1	Ubiquitination of Epsin 1	Important for clathrin-mediated endocytosis of virus	Chen and Zhuang, 2008
Entry/fusion	NEDD4	Ubiquitination of IFITM3	Promotes viral fusion	Chesarino et al., 2015
	/	*S*-palmitoylation of IFITM3	Decreases viral fusion	Yount et al., 2010
Entry/uncoating	Itch	Ubiquitination of M1	Ensure efficient uncoating	Su et al., 2013
	HDAC6	Ubiquitination	Ensure efficient uncoating	Banerjee et al., 2014
Entry/nuclear import of vRNPs and vRNPs	/	Phosphorylation of NP at S9 and Y10	Prevents nuclear import of NP	Zheng et al., 2015
components	HDAC6/8	Acetylation of Hsp90 by inhibiting HDAC6/8 activity	Decreases viral polymerases nuclear accumulation	Panella et al., 2016
	HDAC1	Decreased acetylation level of NP by HDAC1	Facilitates NP nuclear import	Chen et al., 2017
	TRIM14	Ubiquitination of NP by TRIM14	Decreases NP nuclear import	Wu et al., 2019
Transcription	GCN5	Acetylation of NP at K90	Increases polymerase activities	Hatakeyama et al., 2018
	PCAF	Acetylation of NP at K31	Decreases polymerase activities	Hatakeyama et al., 2018
	/	Phosphorylation of NP at S165	Decreases transcription and replication	Turrell et al., 2015
	/	Phosphorylation of NS1 at T80	Decreases NS1-NP interaction and subsequent viral transcription	Zheng et al., 2017
Viral RNA synthesis	/	Ubiquitination	Promotes virus RNA synthesis	Widjaja et al., 2010
vRNPs assembly	/	Phosphorylation of NP at S407 and S413	Inhibits vRNPs assembly	Mondal et al., 2015
	PKCα	Phosphorylation of NP at S165 and S407	Impairs vRNPs assembly	Mondal et al., 2017
	/	Hyper-acetylation of NP at K77 and K229	Disturbs vRNPs formation	Giese et al., 2017
	CDC25B	Dephosphorylation of NP	Enhances NP self-oligomerization and NP nuclear export	Cui et al., 2018
Viral RNA replication	TRIM41	Ubiquitination of NP	Limits virus infection	Patil et al., 2018
	TRIM22	Ubiquitination of NP	Limits virus infection	Di Pietro et al., 2013
	USP11	Deubiquitinating on K184 of NP	Inhibits viral RNA replication	Liao et al., 2010
	CNOT4	Ubiquitinating NP	Enhances replication by serving as competing enzymes with USP11	Lin et al., 2017
	/	Phosphorylation of PA at T157	Associates with PA proteolytic activity and vRNA replication	Perales et al., 2000
	PKM2	Phosphorylation of PA	Conversion of the function of RdRp from transcriptase to replicase	Miyake et al., 2017
	PKCα	Phosphorylation of NS1 at S42	Attenuates viral replication	Hsiang et al., 2012
	CDK, ERK	Phosphorylate NS1 at T215	Promotes viral replication	Hale et al., 2009
	PDPK1	Phosphorylation of Akt at T308	Promotes viral replication	Kuss-Duerkop et al., 2017
	HDM2	NEDDylation of PB2	Reduces PB2 stability and viral replication	Zhang et al., 2017
Nuclear export of vRNPs and vRNPs components	/	Phosphorylation of RanBP3 at S58	Associates with the normal vRNPs nuclear export	Predicala and Zhou, 2013
	AIMP2	SUMOylation of M1 at K242	Promotes M1-mediated viral RNPs nuclear export	Gao et al., 2015
	/	Phosphorylation of M1at Y132	Promotes M1-mediated viral RNPs nuclear export	Wang et al., 2013
	/	Phosphorylation of NP at Y296	Obstructs nuclear export of vRNPs by inhibiting NP binding to CRM1	Zheng et al., 2015
	/	Phosphorylation of MLC	Promotes RNP complex nuclear export	Haidari et al., 2011
	/	Phosphorylation of NP at T188	Impedes nuclear export signal 2-dependent NP nuclear export	Li et al., 2018
Cytoplasmic transport of vRNPs	HDAC6	Acetylation of α-tubulin	Downregulates the trafficking of viral RNPs to the host cell plasma membrane	Husain and Cheung, 2014
Assembly/packaging	/	Ubiquitination of M2 at K78	Facilitates the packaging of the viral genome into virus particles	Su et al., 2018
	/	Palmitoylation of HA	Correlates with assembly and infectious influenza virus particles formation	Chen et al., 2005
Virus budding/release	/	SUMOylation of M1 at K242	Promotes virus release by facilitating M1-vRNPs complex formation	Wu et al., 2011
	/	Acetylation of microtubules	Increases virus release	Husain and Harrod, 2011
	/	Hyper-acetylation of NP at K229	Impairs viral particle release	Giese et al., 2017

*^a^“/” indicates that no precise host factors have yet been found to be associated with post-translational modifications during this process. ^b^A representative reference is listed. Please see the text for more details.*

### PTMs Involved in IAV Binding and Internalization

The initial viral entry step is virion attachment to the host cell through the binding of HA with the sialic acid receptor, which is regulated by species-specific sialic acid linkages. Usually, the HA protein of avian IAV preferentially binds to sialic acids linked to galactose by an α-2, 3-linkage receptor (Sia α2, 3 Gal), whereas the HA protein of human IAV shows a high affinity for Sia α2, 6 Gal receptor (Weis et al., 1988; Gamblin et al., 2004; Stevens et al., 2006). Interestingly, the preference for sialic acids with different terminal linkages and the affinity for sialic acid partially stem from the N-linked glycosylation status of HA (de Vries et al., 2010). Moreover, the extent of HA glycosylation may play a role in the cross-species transmission of influenza virus (Herfst et al., 2012). However, although it is generally accepted that the sialic acid receptor is the main receptor used by IAV for entry, additional host factors, such as Annexin V (Huang et al., 1996), C-type lectins (Londrigan et al., 2011), and 6-sulfo sialyl Lewis X receptors (Gambaryan et al., 2008), have also been proposed as alternative host receptors used by IAV for successful attachment and entry into the target cells.

Viral attachment to the host cell triggers virion internalization via Clathrin-mediated endocytosis (Roy et al., 2000; Rust et al., 2004; Chen and Zhuang, 2008), Clathrin-independent endocytosis (Nunes-Correia et al., 2004), or macropinocytosis (Sieczkarski and Whittaker, 2002; Rust et al., 2004; de Vries et al., 2011). The internalization of IAV through Clathrin-mediated endocytosis involves Dynamin (Roy et al., 2000) and requires the adaptor protein Epsin 1 (Chen and Zhuang, 2008). The UIM of Epsin 1 plays a critical role in the Clathrin-mediated endocytosis of influenza virus (Chen and Zhuang, 2008) (Table 1). Non-structural protein 1 (NS1) can interact with Akt, a core intracellular survival regulator and the major effector molecule of phosphoinositide 3-kinase (PI3K). Suppression of Akt kinase activity decreases the phosphorylation levels of NS1 and glycogen synthase kinase 3 (Akt substrate), leading to reduced viral internalization efficiency and viral replication (Hirata et al., 2014) (Table 1).

### PTMs Involved in IAV Endosome Trafficking and Fusion

Upon influenza virus internalization by either the endocytosis or macropinocytosis uptake pathway, endosomal trafficking from early endosomes to late endosomes begins. IFITM3 can localize to endosomes, acting as a viral fusion inhibitor and exerting a broad-spectrum antiviral activity. However, the E3 ubiquitin ligase NEDD4 can alleviate IFITM3-mediated inhibition of fusion by decreasing IFITM3 expression via ubiquitination (Chesarino et al., 2015) (Table 1). In contrast, *S*-palmitoylation promotes the antiviral activity of IFITM3, mainly through manipulating its clustering in membranes and blocking influenza virus fusion (Dawson and Mehle, 2018).

### PTMs Involved in Uncoating and Nuclear Import of IAV vRNPs and vRNP Components

**FIGURE 2:**
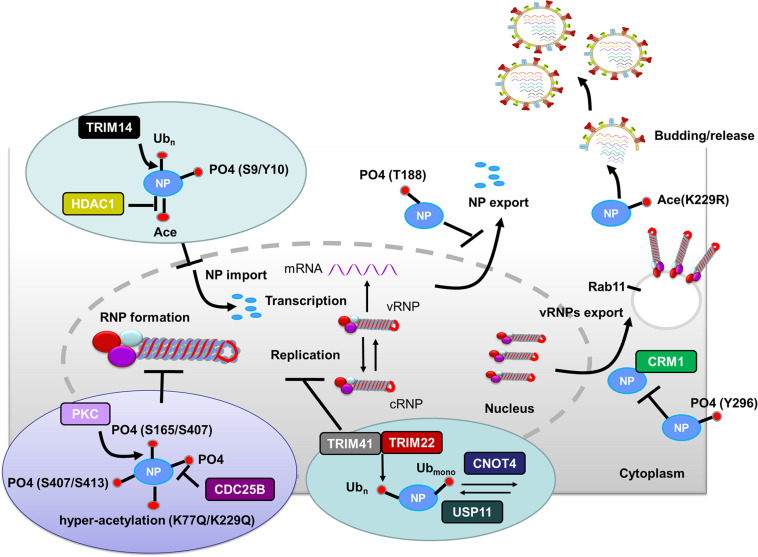
Role of PTMs in regulating NP functions. Phosphorylation of NP at S9 and Y10 and ubiquitination of NP by TRIM14 prevent NP nuclear import. HDAC decreases NP acetylation and facilitates NP nuclear import. Phosphorylation of NP at S407 and S413 inhibits the assembly of influenza virus replication machinery. Mimicking hyperacetylation of NP at K77Q and K229Q disturbs vRNPs formation. Human PKCα blocks NP oligomerization through phosphorylating NP at S165 and S407. In contrast, CDC25B facilitates the dephosphorylation of NP, leading to enhanced NP self-oligomerization. TRIM41 and TRIM22 impede viral replication through ubiquitinating NP. The cellular deubiquitinating enzyme USP11 inhibits vRNA replication through deubiquitinating NP K184; CNOT4 competes with USP11 in the regulation of NP ubiquitination and enhances vRNA replication. Phosphorylation of NP Y296 impedes vRNPs nuclear export via inhibiting the interaction of NP with the export factor CRM1. Phosphorylation of NP T188 impedes NP nuclear export. Exported vRNPs associate with Rab11-positive vesicles and traffic from the cytoplasm to the apical cell surface for virion budding. NP acetylation plays a role in virion release. Abbreviations: Ub, ubiquitin and ubiquitination; PO4, phosphorylation; Ace, acetylation; vRNP(s), viral ribonucleoprotein(s); mRNA, messenger RNA; PKCα, protein kinase C α; CDC, cell division cycle; TRIM, tripartite motif; HDAC, histone deacetylase; USP11, ubiquitin–proteasome system protein 11; CNOT4, Ccr4-Not transcription complex subunit 4; CRM1, chromosome region maintenance 1 protein homolog.

After HA-mediated membrane fusion occurs in late endocytic vacuoles, the viral capsids are released from the cytosolic surface of endosomes, the vRNPs and the M1 protein then dissociate from each other and disperse in the cytosol, and the vRNPs are imported into the nucleus through nuclear pore complexes (Martin and Helenius, 1991; O’Neill et al., 1995). During this process, IAV takes advantage of the host cellular ubiquitin-dependent aggresome formation and disassembly machinery (Banerjee et al., 2014). The unanchored ubiquitin chains, which are packaged into virions, can facilitate viral uncoating by recruiting histone deacetylase (HDAC) 6 to viral fusion sites via the zinc-finger ubiquitin-binding domain (ZnFUBP) of HDAC6 (Banerjee et al., 2014). Itch, an E3 ubiquitin ligase, also enables efficient uncoating via ubiquitinating M1, thereby facilitating vRNPs release from the endosomes (Su et al., 2013) (Table 1 and Figure 1). Released vRNPs then move toward the nucleus to complete the last step of viral entry (vRNPs nuclear import) and begin viral transcription. Currently, very few PTMs have been shown to directly regulate the localization of viral polymerases. However, the inhibition of HDAC6/8 activity by HDAC-inhibitor MC1568 resulted in the early acetylation of HSP90 and led to a decrease in viral polymerases nuclear accumulation and a subsequent attenuated viral replication (Panella et al., 2016) (Table 1). Nuclear import of the vRNP components, such as NP, is finely regulated by phosphorylation, SUMOylation, and acetylation. Phosphorylation at S9 or Y10 in the nuclear localization signal (NLS) of NP prevents its nuclear import by regulating the association of this protein with its nuclear import receptors, such as Importin-α (Zheng et al., 2015) (Figure 2). The NP-interacting protein TRIM14 stimulates K48-linked ubiquitination and proteasomal degradation of NP, leading to a reduced efficiency in NP transportation from the cytoplasm to the nucleus and a subsequent inhibition of viral replication (Wu et al., 2019) (Table 1 and Figure 2). Meanwhile, HDAC1 interacts with NP, resulting in a decreased level of NP acetylation, thus facilitating viral replication through promoting the nuclear import of NP and suppressing the TBK1-IRF3 signal pathway (Chen et al., 2017) (Table 1 and Figure 2).

## PTMs Involved in the Viral Transcription and Replication of IAV

The replication of IAV RNA requires an intricate regulatory network composed of both viral and cellular proteins. IAV uses distinctly different strategies for transcription and replication (Barrett et al., 1979). For transcription, 5′-capped oligonucleotides derive from cellular mRNAs by cap-snatching are used as primers. In contrast, no primers are needed for replication. The vRNA template is directly copied to form a full-length positive-stranded RNA (cRNA), which serves as the template for vRNA synthesis (Hay et al., 1982). Moreover, another major difference between transcription and replication is that influenza virus RNA replication requires newly synthesized viral proteins. PTMs of viral proteins and host factors play important roles in regulating influenza virus replication. Here, we summarize the PTMs involved in the process of producing viral transcripts and new copies of the viral genome.

### PTMs Involved in IAV Transcription

Influenza virus uses a unique strategy for priming transcription, referred to as “cap-snatching,” where short 5′-fragments containing 7-methylG caps are cleaved from host messenger RNAs (mRNAs) and used to prime the synthesis of viral mRNAs (Plotch et al., 1981). To complete the cap-snatching process, the transcribing polymerases must gain access to the nascent host mRNA through binding to cellular RNA polymerase II (RNAPII) and selectively recognizing PTM patterns in its C-terminal domain (CTD) (i.e., phosphorylated CTD) (Engelhardt et al., 2005).

The acetylation and phosphorylation of NP are actively involved in regulating influenza viral transcription. The host acetyltransferase GCN5 can acetylate NP K90, leading to increased IAV polymerase activities, whereas acetylation of NP K31 mediated by PCAF decreases polymerase activities (Hatakeyama et al., 2018) (Table 1). The phosphorylation of NP can stimulate viral transcription, and NP must undergo specific conformational changes to ensure efficient transcriptional activity (Kamata and Watanabe, 1977). As a PB1-interacting protein, the E3 ubiquitin ligase TRIM32 directly limits viral infection in mice by targeting PB1 for ubiquitination, which downregulates viral polymerase activity (Fu et al., 2015). This intrinsic antiviral defense strategy provides a novel target for drugs to prevent and treat influenza. PKCα is a multifunctional effector in influenza virus-induced signaling cascades. PKCα mediates the phosphorylation of PB1 and NS1, consequently contributing to the activity of vRNA polymerase complexes (Mahmoudian et al., 2009). Moreover, the phosphorylation of NS1 T80 reduces IAV replication both *in vitro* and *in vivo* through two mechanisms: (1) decreasing NS1-NP interactions, leading to impaired vRNPs-mediated viral transcription, and (2) reducing the binding affinity between NS1 and RIG-I, resulting in the virus being unable to inhibit IFN production (Zheng et al., 2017).

### PTMs Involved in IAV vRNA Synthesis

Influenza virus replication depends on the ubiquitin-proteasome system. Several studies have preliminarily reported an important role of ubiquitination in virus entry (Khor et al., 2003; Chen and Zhuang, 2008) and vRNA synthesis (Konig et al., 2010). However, there remains a lack of direct evidence for this phenomenon. Widjaja et al. used both proteasome inhibitors (MG132 and bortezomib) and the E36ts20 hamster cell line harboring a temperature-sensitive mutation in the E1-activating enzyme to investigate the detailed role of the ubiquitin-proteasome system in IAV replication cycle (Widjaja et al., 2010). Their results indicate that ubiquitination efficiently promotes vRNA synthesis and subsequent protein expression (Widjaja et al., 2010).

### PTMs Involved in IAV Viral Genome Replication and vRNPs Assembly

The vRNPs complex contains the viral polymerase, genomic RNA, and NP. The NP protein exists in an oligomeric form consisting of multiple copies, which bind to and coat the viral RNA genome. Before vRNPs complex assembly begins, the virus must complete primary transcription and transition to genome replication. During the vRNPs assembly process, an NP monomer is initially recruited through direct interaction with viral polymerase. After binding to the nascent 5’ end of the viral genome, monomeric NP oligomerizes along the length of the genomic RNA via NP-NP homo-oligomerization (Vreede et al., 2004; Ye et al., 2006; Tao and Ye, 2010; Arranz et al., 2012; Moeller et al., 2012). Phosphorylation of NP inhibits the assembly of influenza virus replication machinery. Mechanistically, two phosphorylation sites (S407 and S413) on the opposite sides of the NP-NP interface block homotypic interactions and destroy the balance between NP monomers and oligomers, thus maintaining the monomeric form of NP (Mondal et al., 2015) (Figure 2). Human PKCα impairs vRNPs assembly through phosphorylating NP at the tail loop-groove interface (major targets: S165 and S407), thus blocking NP oligomerization and vRNPs formation in cells (Mondal et al., 2017) (Table 1 and Figure 2). In addition, mimicking hyperacetylation of NP at K77Q and K229Q disturbs vRNPs formation and severely diminishes viral polymerase activity (Giese et al., 2017) (Table 1 and Figure 2). S165 is present within the NP homo-oligomerization domain. It was proposed that reversible phosphorylation of NP S165 decreases NP oligomerization prior to vRNPs assembly and inhibits mRNA and vRNA accumulation, resulting in subsequent attenuation of viral growth in Madin–Darby bovine kidney (MDBK) cells (Turrell et al., 2015). CDC25B, a member of the CDC25 phosphatase family, facilitates the dephosphorylation of NP, leading to enhanced self-oligomerization and nuclear export of NP along with increased viral polymerase activity and vRNA production (Cui et al., 2018) (Table 1 and Figure 2).

The phosphorylation of other proteins also plays a role in influenza virus replication. For example, the phosphorylation of PA is highly associated with its proteolytic activity, which decreases the expression levels of both PA and its co-expressed proteins (Hara et al., 2001, 2003). Casein kinase II can target 11 potential phosphorylation sites on PA. Among these sites, a T157A mutation was found to cause defective phosphorylation and almost completely abrogate proteolysis activity, leading to sharply reduced vRNA replication (Perales et al., 2000) (Table 1). Pyruvate kinase M2 (PKM2), an important enzyme for ATP generation (Altenberg and Greulich, 2004), can interact with PA. PKM2 positively regulates viral replication possibly through phosphorylation of PA, which leads to RdRp switching its function from a transcriptase to a replicase (Miyake et al., 2017). Furthermore, phosphorylation of NS1 S42 catalyzed by PKCα attenuates human IAV replication (Hsiang et al., 2012). Cyclin-dependent kinases (CDKs) and extracellular signal-regulated kinases (ERKs) can phosphorylate NS1 T215 and contribute to efficient virus replication (Hale et al., 2009). Moreover, NS1 can induce G_0_/G_1_ cell cycle arrest by interfering with the RhoA-pRb signaling cascade through downregulating the phosphorylation level of cell cycle regulator pRb, thus providing favorable conditions for viral protein accumulation and replication (Jiang et al., 2013).

The host kinase mechanistic target of rapamycin (mTOR), a cellular regulator of protein synthesis, growth, and motility, is coopted by influenza virus to promote infection (Konig et al., 2010; Mata et al., 2011). To maximize viral replication during the late stages of infection, influenza virus differentially activates the signaling pathways of mTOR complex 1 and 2 (mTORC1 and mTORC2, respectively) that are two functionally distinct multiprotein complexes (Kuss-Duerkop et al., 2017). Influenza virus replication and HA can ensure efficient viral replication by enhancing mTORC1 activation through PDPK1-mediated phosphorylation of Akt at T308. Influenza virus M2 then further promotes viral replication by downregulating the expression of REDD1 (an mTORC1 inhibitor) to support mTORC1 activation. Finally, NS1 promotes mTORC2-mediated phosphorylation of Akt at S473 and subsequently enhances cell apoptosis (Kuss-Duerkop et al., 2017).

Ubiquitination contributes to proteasomal degradation, and all the proteins in the influenza virus replication machinery are ubiquitinated (Kirui et al., 2016). Most of the NP ubiquitinations are associated with viral replication. TRIM protein superfamily members are emerging as crucial antiviral effectors, which often rely on their E3 ubiquitin ligase activities to restrict influenza viral replication. TRIM41 acts as an intrinsic host restriction factor for IAV, limiting viral infection by directly binding to NP, leading to its ubiquitination and subsequent proteasomal degradation (Patil et al., 2018) (Figure 2). TRIM22, another NP-interacting protein, is highly upregulated in human alveolar epithelial A549 cells and also acts to restrict IAV replication by degrading viral NP via its ubiquitination (Di Pietro et al., 2013) (Figure 2).

Deubiquitination of NP can also affect influenza viral replication. The cellular deubiquitinating enzyme USP11 inhibits vRNA replication through deubiquitinating NP K184 to lower the NP binding affinity for cRNA (Liao et al., 2010). CNOT4, a cellular ubiquitin ligase, competes with USP11 in the regulation of NP ubiquitination, and thereby enhancing vRNPs activity and vRNA replication (Lin et al., 2017) (Table 1 and Figure 2). Thus, NP ubiquitination has both proviral and antiviral roles, and the ubiquitination and deubiquitination of NP must be properly balanced for efficient influenza infection.

NEDDylation is another important modification that regulates protein activity and protein–protein interactions. It has been shown to play important roles during the life cycles of various viruses, such as human immunodeficiency virus type-1 (HIV-1) and Kaposi’s sarcoma-associated herpesvirus (Esposito et al., 2009; Soucy et al., 2010; Stanley et al., 2012; Hughes et al., 2015). Influenza virus PB2 can be NEDDylated at residue K669 by NEDDylation E3 ligase HDM2, leading to reduced PB2 stability and a subsequent weakened viral replication and virulence in mice (Zhang et al., 2017).

## PTMs Involved in IAV Nuclear Export, Trafficking, and Budding

In the later steps of the influenza viral replication cycle, newly synthesized vRNPs are exported out of the nucleus and trafficked to the budding site at the plasma membrane, where they are assembled into virions and ultimately bud from the infected cell (Lakdawala et al., 2016). At the same time, the influenza virus structural proteins NA, HA, M1, and M2 are processed and accumulate at the virion assembly sites on the plasma membrane. Knowledge of how PTMs contribute to these processes has been growing quickly in recent years. In the following section, we will review the current progress regarding the involvement of PTMs in the later steps of the influenza virus life cycle (Table 1).

### PTMs Involved in the Nuclear Export of IAV vRNPs and vRNP Components

Newly synthesized vRNPs are exported out from the nucleus through the nuclear pore complex, which relies on host cellular export machinery. CRM1 is the major exporting factor responsible for the transport of influenza virus vRNPs from the nucleus to the cytoplasm. During this process, M1 plays an important role in facilitating the interaction between vRNPs and the host cellular export system. Specifically, vRNPs can interact with M1, M1 can interact with nuclear export protein (NEP), and NEP can interact with CRM1 (Paterson and Fodor, 2012). Therefore, M1 likely acts as an adapter protein between vRNPs, NEP, and the host cellular export system. M1 with phosphorylation modification plays a concerted role in promoting the nuclear export of vRNPs during influenza virus infection (Bui et al., 2000). Additionally, AIMP2, an NS1-interacting protein, enhances M1 stability and changes the modification state of M1 K242 from ubiquitinated to SUMOylated, thus promoting M1-mediated vRNPs nuclear export (Gao et al., 2015) (Table 1 and Figure 1). Phosphorylation of M1 Y132 is essential for influenza virus replication, which controls the nuclear import of M1 and the subsequent nuclear export of progeny vRNPs by regulating the interaction between M1 and the nuclear import factor importin-1 (Wang et al., 2013) (Figure 1). Moreover, SUMOylation of M1 also enhances its interactions with vRNPs and promotes the subsequent nuclear export of vRNPs (Wu et al., 2011).

Phosphorylation of NP Y296 obstructs the nuclear export of vRNPs complexes and subsequently impairs virus assembly and polymerase activity through inhibiting the binding affinity of NP to export factor CRM1 (Zheng et al., 2015) (Figure 2). RanBP3 is a Ran-interacting protein that acts as a nuclear export cofactor of CRM1-mediated cargo during influenza virus infection. Phosphorylation of RanBP3 at S58 is associated with normal vRNPs nuclear export, and downregulation of RanBP3 expression impairs the nuclear export of influenza virus vRNPs (Predicala and Zhou, 2013).

The actin and microtubule cytoskeleton also play a critical role in viral replication (Radtke et al., 2006). Actin cytoskeleton contraction and relaxation are predominantly regulated by phosphorylation and dephosphorylation of the regulatory subunit of MLC (Lincoln, 2007). Influenza virus infection activates a series of signaling pathways that induce MLC phosphorylation and actin cytoskeleton remodeling. The inhibition of MLC phosphorylation leads to nuclear retention of influenza vRNPs complexes and a reduction of influenza virus replication, whereas the induction of MLC phosphorylation reverses the inhibitory effects of unphosphorylated MLC on the nuclear translocation of influenza virus vRNPs complexes (Haidari et al., 2011). Nuclear export of vRNPs requires sufficient amounts of HA and NA to be embedded in the plasma membrane, and this process is coordinated in part by PKCα/mitogen-activated protein (MAP) kinase signaling cascade mediated by HA membrane accumulation (Pleschka et al., 2001; Marjuki et al., 2006). Blocking this pathway during influenza virus infection leads to vRNPs nuclear retention, impaired nuclear-export protein (NEP/NS2) function, and concomitant inhibited virus propagation (Pleschka et al., 2001; Marjuki et al., 2006).

The NP protein has an intrinsic ability to be exported from the nucleus to the cytoplasm, and the nuclear export ability of NP is partially regulated by phosphorylation. A lack of NP phosphorylation may block NP nuclear export or allow a rapid reimportation of NP into the nucleus (Neumann et al., 1997). Phosphorylation of NP T188 impedes NES2-dependent NP nuclear export, decreases viral polymerase activity as well as viral replication (Li et al., 2018) (Figure 2).

### PTMs Involved in IAV Protein Trafficking to the Plasma Membrane

Influenza virus assembles at the host cell plasma membrane. Before budding, the newly synthesized vRNPs and viral membrane proteins (HA, NA, and M2) are translocated to the budding site at the plasma membrane via different trafficking systems. To reach the budding site, the vRNPs mainly use the microtubule network and vesicular transport system (Hutchinson and Fodor, 2013), whereas HA, NA, and M2 use the ER-Golgi secretory network. Microtubules are the largest cytoskeleton component, and they play a crucial role in a variety of cellular functions, including intracellular transport, organelle positioning, cell shape and motility, and centrosome and cilium formation (Janke and Bulinski, 2011). To accomplish such diverse functions, microtubules associate with motor and non-motor proteins and undergo posttranslational modifications such as acetylation and detyrosination (Janke and Bulinski, 2011). During influenza virus infection, downregulation of tubulin deacetylase and HDAC6 activity enhances α-tubulin acetylation, subsequently facilitating virion release (Husain and Harrod, 2011). Mechanistically, HDAC6 downregulates the trafficking of vRNPs to the host cell plasma membrane via reducing the amount of acetylated microtubules (Husain and Cheung, 2014) (Table 1).

### PTMs Involved in IAV Assembly, Budding, and Release

M2 ubiquitination plays an important role in influenza virus replication through facilitating the packaging of viral genome into virus particles and coordinating the timing of virus-induced host cell apoptosis and autophagy (Su et al., 2018) (Figure 1). Moreover, ubiquitination-deficient mutant M2 K78R, had lower infectivity because it produced mainly defective virion particles that either lacked vRNPs or contained smaller amounts of internal viral components (Su et al., 2018) (Figure 1).

Influenza A viruses assembly requires the coordinated localization of different viral components at virus budding sites. M1 can interact with vRNPs, thus playing a key role in influenza virus assembly (Schmitt and Lamb, 2005). M1 SUMOylation at K242 is required for efficient interaction between M1 and vRNPs to form the M1-vRNP complex, thus contributing to viral maturation and assembly (Wu et al., 2011). Viruses carrying a SUMO-deficient M1 produce fewer infectious particles and form virions with more elongated or filamentous structures, indicating that a lack of M1 SUMOylation impairs viral morphogenesis (Wu et al., 2011). M1 also interacts with the viral envelope proteins HA, NA, and M2 via their cytoplasmic tails. Palmitoylation of HA at the conserved carboxy-terminal of the cytoplasmic tail is required for efficient M1 recruitment and correlates with the ability to form infectious influenza virus particles (Zurcher et al., 1994; Chen et al., 2005). Acetylation is another PTM known to regulate protein function. Enhanced acetylation of microtubules increases IAV release from infected cells (Husain and Harrod, 2011). Furthermore, the growth of NP acetylation-deficient K229R mutant was severely attenuated in various cell types due to impaired virion release (Giese et al., 2017) (Figure 2).

## Role of PTMs in the Antiviral Innate Immune Response to IAV

Innate immunity acts as the first-line defense of host cells in restricting IAV replication (Iwasaki and Pillai, 2014). During virus infection, the pattern recognition receptors (PRR) recognize pathogen-associated molecular patterns (PAMPs) in the invading viruses, leading to the activation of innate immune signaling cascades and the subsequent production of proinflammatory cytokines (Iwasaki and Pillai, 2014). PTMs are involve in regulating innate immune signaling, and many viruses have evolved various mechanisms to usurp PTMs to counteract this host defense response (Calistri et al., 2014). For influenza virus, the activities of the immune-regulatory proteins, including NS1 (Nogales et al., 2018) and the accessory proteins PA-X (Levene and Gaglia, 2018; Nogales et al., 2018) and PB1-F2 (Cheung et al., 2020), are finely modulated by various PTMs to antagonize host innate immune responses.

### Role of PTMs in IAV NS1 Antagonism of Host Antiviral Innate Immune Responses

**FIGURE 3:**
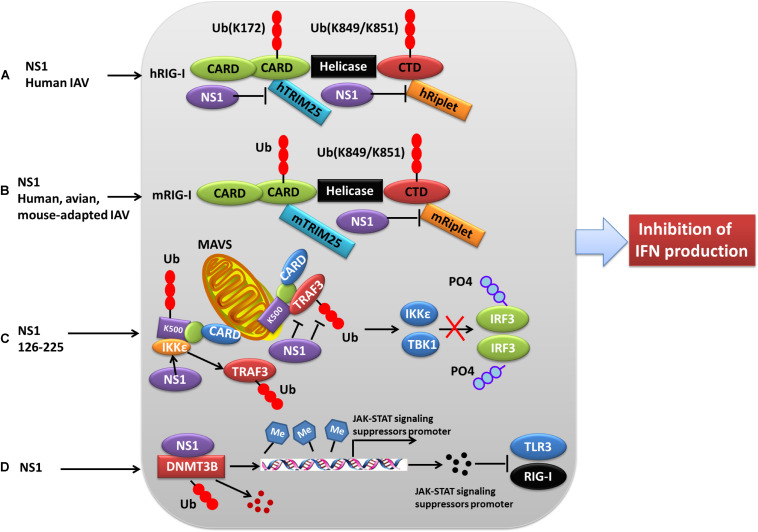
Role of PTMs in NS1 antagonism of host interferon responses. **(A)** NS1 from human IAV strains can interact with human TRIM25 and Riplet, blocking them from mediating the ubiquitination of the vRNA sensor RIG-I CARD, and this eventually leads to the inhibition of IFN production. **(B)** NS1 proteins from avian, human, and mouse-adapted influenza viruses can inhibit RIG-I signaling in mouse cells only through binding to and blocking mouse Riplet. **(C)** The NS1 C-terminal effector domain (NS1 126–225) associates with TRAF3, promoting the disassociation of TRAF3 from MAVS and leading to a decreased level of K63-linked ubiquitination of TRAF3. Moreover, NS1 126–225 facilitates the recruitment of IKKε to MAVS, resulting in TRAF3 release from the mitochondria and impaired IRF3 phosphorylation. **(D)** NS1 interacts with cellular DNMT3B, and the resulting NS1-DNMTcomplex is translocated from the nucleus to the cytosol where it undergoes K48-linked polyubiquitination of DNMT3B. This leads to demethylation of the methylated promoters of JAK-STAT signaling suppressors and the subsequent expression of associated proteins, ultimately resulting in the inhibition of TLR3 and RIG-I. Ub, ubiquitin and ubiquitination; PO4, phosphorylation; TRIM, tripartite motif; RIG-I, retinoic acid-induced gene 1 protein; CARD, caspase recruitment domain; DNMT3B, DNA methyltransferase; TRAF3, TNF receptor-associated factor 3.

Production of type I IFNs, a family of anti-viral cytokines, is an integral component of the host innate immunity defense system, which play a critical role in inhibiting the accomplishment of the virus life cycle and impeding virus dissemination *in vivo* (Garcia-Sastre et al., 1998). Influenza virus NS1 counters the IFN antiviral responses mainly through sequestering RNAs that are sensed to trigger signaling cascade activation, or interfering with the vRNA sensors themselves, particularly RIG-I and its activator TRIM25. Generally, ubiquitination by TRIM25 of the vRNA sensor RIG-I CARD with K63-linked poly-ubiquitin chains leads to IFN production. However, this process can be blocked through the interaction of NS1 E96 and E97 with TRIM25 coiled-coil-PRYSPRY domain (Gack et al., 2009). Mechanistically, NS1 binding affects the correct positioning of the TRIM25 PRYSPRY domain that is required for substrate ubiquitination, thus suppressing RIG-I ubiquitination and subsequent downstream signaling (Koliopoulos et al., 2018). Moreover, NS1 targets TRIM25 in a species-specific manner to inhibit RIG-I ubiquitination and antiviral IFN production (Rajsbaum et al., 2012) (Figure 3). Specifically, NS1 derived from human influenza viruses binds to and suppresses both human TRIM25 and Riplet-mediated RIG-I CARD and CTD ubiquitination (Figure 3A). In contrast, NS1 from avian, human, and mouse-adapted influenza viruses inhibits RIG-I signaling in mouse cells only through binding to and blocking mouse Riplet (Figure 3B) (Rajsbaum et al., 2012). These findings for NS1 partly explain the host adaptation ability of influenza virus.

NS1 can also antagonize the IFN-β response via NS1 126-225 binding to the TRAF domain of TRAF3, which suppresses TRAF3 K63-linked ubiquitination (Qian et al., 2017) (Figure 3C). Another mechanism by which NS1 antagonizes the host IFN response during influenza virus infection is modulation of JAK-STAT signaling (Liu S. et al., 2019). Mechanistically, the newly expressed NS1 migrates to the nucleus where it interacts with DNMT3B, and then the NS1-DNMTcomplexes are transported from the nucleus to the cytosol where they undergo K48-linked polyubiquitination of the DNMT3B. This leads to a demethylation of the methylated promoters for the JAK-STAT signaling suppressor, thus contributing to the subsequent inhibition of TLR3 and RIG-I activity (Liu S. et al., 2019) (Figure 3D).

**FIGURE 4:**
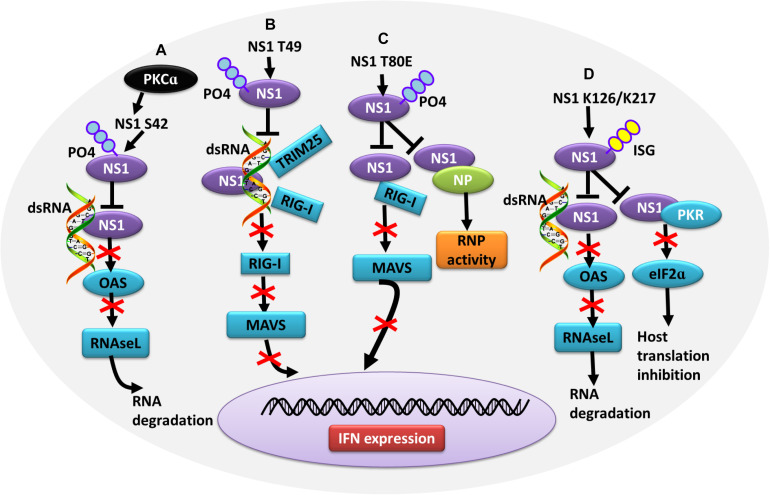
Role of PTMs in alleviating NS1 antagonism of the host antiviral response. **(A)** Phosphorylation of NS1 at S42 by PKCα can block NS1 binding to dsRNA, alleviating NS1-induced inhibition of OAS activation and RNA degradation. **(B)** Phosphorylation of NS1 at T49 leads to decreased interaction with dsRNA, TRIM25, and RIG-I, resulting in defective suppression of IFN production. **(C)** Phosphorylation of NS1 at T80 reduces the binding affinity between NS1 and RIG-I, preventing NS1 from inhibiting IFN production. **(D)** Herc5-mediated ISGylation of NS1 K126 and K217 inhibits RNA sequestration, alleviating NS1-induced inhibition of OAS activation and RNA degradation. ISGylation of NS1 K126 and K217 impair the NS1 interaction with PKR, which antagonizes NS1-mediated inhibition of host translation. Ub, ubiquitin and ubiquitination; ISG, ISGylation; PKCα, protein kinase C α; OAS, oligo (A) synthetase; TRIM, tripartite motif; PKR, protein kinase R.

In contrast, some PTMs negatively regulate the ability of NS1 to counteract the host IFN response, thus contributing to virus attenuation. During the later stages of influenza virus infection, the phosphorylation of NS1 at T49 leads to a reduced interaction with double-stranded RNA (dsRNA), TRIM25, and RIG-I, resulting in a defective suppression of IFN induction (Kathum et al., 2016) (Figure 4B). Moreover, as the infectious cycle progresses, dynamic phosphorylation of NS1 at T49 may alter NS1 activity (Hsiang et al., 2012). NS1 phosphorylated at T80 also fails to inhibit IFN production due to its reduced binding affinity with RIG-I, which impedes influenza virus replication (Zheng et al., 2017) (Figure 4C). Additionally, phosphorylation of NS1 at S42 by PKCα can block the binding of NS1 to dsRNA (Hsiang et al., 2012) (Figure 4A). NS1 is SUMOylated at residues K70 and K219. A non-SUMOylatable form of NS1 failed to inhibit IFN expression, and an excessive of NS1 SUMOylation also exerted a negative effect on its IFN-blocking function. Therefore, it is likely that an optimal level of NS1 SUMOylation is needed to endow NS1 with maximal activity of blocking IFN (Xu et al., 2011; Santos et al., 2013). ISGylation also limits influenza virus virulence. The IFN-induced ISG15 conjugation system can exert antiviral activity by conjugating ISG15 to NS1 in infected cells, thereby antagonizing various functions of NS1 and hence virus replication. ISG15 modification of NS1 K41 by the host E3 ligase Herc5 contributes to antagonizing IFN-β response and disrupting the interaction of the NS1 RNA-binding domain (RBD) with importin-α (Zhao et al., 2010). Furthermore, Herc5-mediated ISGylation of NS1 K126 and K217 inhibits NS1 homodimerization, prevents RNA sequestration, impairs NS1 interaction with PKR, and interferes with the inhibitory effect of NS1 on virus-induced IFN-β expression (Tang et al., 2010) (Figure 4D).

Influenza A viruses infection can activate the NLRP3 inflammasome, resulting in the production of IL-1β, a major mediator of inflammation (Guo et al., 2015). However, this NLRP3 inflammasome-mediated IL-1β expression can be hindered by the C terminus of pandemic influenza virus NS1, which inhibits speck formation and ubiquitination of the inflammasome adaptor ASC (Park et al., 2018). Moreover, the ubiquitination sites K110 and K140 in the C terminus of porcine ASC are critical for both ASC ubiquitination and subsequent NLRP3 inflammasome-mediated IL-1β production (Park et al., 2018). Furthermore, NS1 counteracts the host NF-κB-mediated antiviral response by disrupting IKK function (Gao et al., 2012). More specifically, through its interaction with IKKα/IKKβ, NS1 not only blocks IKKβ-mediated IkBα phosphorylation and degradation in the cytoplasm but also impairs IKK-mediated histone H3 phosphorylation, which are crucial for triggering the rapid expression of NF-κB-regulated genes in the nucleus (Gao et al., 2012).

### Role of PTMs in Viral Resistance to Host IFN Responses Mediated by Other IAV Proteins

The antiviral functions of other influenza virus proteins, including HA, PA, PA-X, and PB1-F2, are also regulated by PTMs. HA promotes the phosphorylation and polyubiquitination of IFNAR1, leading to an efficient degradation of IFNAR1 and attenuation of the type I IFN-induced antiviral signaling pathway (Xia et al., 2016). Furthermore, HA induces the degradation of type II IFN receptor 1 (IFNGR1), as well as of IFNAR1, resulting in the impairment of the cellular response to both types I and II IFNs (Xia et al., 2018). Mechanistically, the phosphorylation and ubiquitination of IFNGR1 and IFNAR1 mediated by the cellular kinase CK1α contribute to the HA-induced elimination of type I and type II IFN receptors (Xia et al., 2018).

Polymerase acidic protein can block the activation of IFN-β signaling pathway through the interaction with IRF3 (Yi et al., 2017). Mechanistically, through the endonuclease activity of its N terminus, PA interacts with IRF3 to suppress its phosphorylation, dimerization, and subsequent nuclear translocation and activation (Yi et al., 2017). The accessory proteins PA-X and PB1-F2 also play a role in regulating the host innate immune response. PA-X is a fusion protein that incorporates the N-terminal 191 amino acids PA with a short C-terminal sequence (either 61 or 41 codons) encoded by an overlapping open reading frame (ORF; “X-ORF”) in segment 3 that is accessed by +1 ribosomal frameshifting (Jagger et al., 2012). This small protein acts to decrease the virulence of various influenza viruses through modulating the host innate immune response mainly through its host shutoff activity (Hu et al., 2018; Levene and Gaglia, 2018). Mechanistically, the NatB-mediated N-terminal acetylation of PA-X contributes to this host shutoff activity and viral polymerase activity (Oishi et al., 2018). PB1-F2, another accessory protein, is a 90-amino acid protein that is encoded as an internal open reading frame on the PB1 gene of some influenza viruses. It contributes to the pathogenesis and comorbidity of influenza viruses mainly through manipulating apoptosis and innate immune responses, and enhancing the secondary bacterial infection (Cheung et al., 2020). Loss of PB1-F2 ubiquitination enhances PB1-F2 stability, polymerase activity, and IFN antagonism (Kosik et al., 2015).

### Direct PTMs on Host Innate Immune-Related Proteins

In addition to modification of influenza virus proteins, modification of host proteins also acts as a major mechanism of the host cellular response to control influenza virus infection. Various PTMs are directly involved in regulating the function of several crucial innate immune system proteins, such as IFITM3, TRIM28 (also known as KAP1), MAVS, and NEMO.

IFITM3 is a host antiviral restriction factor that limits cellular infection with multiple notable viral pathogens and is especially crucial for the innate immune response against influenza virus (Yount et al., 2010; Huang et al., 2011). The antiviral activity of IFITM3 is post-translationally regulated by various PTMs, particularly *S*-palmitoylation, ubiquitination, and monomethylation. S-palmitoylation controls IFITM3 clustering in membranes and contributes to its full antiviral activity against influenza virus (Yount et al., 2010). Ubiquitination of IFITM3 by the E3 ubiquitin ligase NEDD4 results in decreased IFITM3 expression and enhanced influenza viral infection (Chesarino et al., 2015). Additionally, IFITM3 K88 can be monomethylated (IFITM3-K88me1) by lysine methyltransferase SET7, resulting in a reduction in IFITM3 antiviral activity. To support efficient infection, IAV upregulates IFITM3 K88me1 by promoting the interaction of IFITM3 with SET7 (Shan et al., 2013). Interestingly, the ubiquitination of IFITM3 K88 can also inhibit IFITM3 antiviral activity. Therefore, the inhibition of these two negative regulatory modifications is speculated to increase antiviral activity (Yount et al., 2012). In contrast, the histone demethylase LSD1 can limit replication of RNA viruses through demethylating and activating IFITM3. However, influenza virus can inactivate IFITM3 by triggering IFITM3 methylation via promoting its disassociation from LSD1 (Shan et al., 2017).

TRIM28 has recently been implicated in contributing to inflammatory cytokine production during influenza virus infection (Domingues et al., 2015; Krischuns et al., 2018; Schmidt et al., 2019). Notably, the loss of SUMOylated TRIM28 results in an increased IFN-mediated antiviral response through the canonical components of dsRNA-sensing pathways, such as RIG-I, MAVS, TBK1, and JAK-STAT (Domingues et al., 2015; Schmidt et al., 2019). Phosphorylation of TRIM28 S473 leads to enhanced expression levels of IFN-β, IL-6, and IL-8 during infection with highly pathogenic avian influenza viruses, such as H7N7, H7N9, and H5N1 (Krischuns et al., 2018).

Mitochondrial antiviral-signaling protein, a key mediator of IFN signaling, plays a critical role in host innate immunity against RNA viruses. PTMs of MAVS, such as K63-linked ubiquitination or phosphorylation, play a critical role in activating IFN signaling (Liu et al., 2015; Liu B. et al., 2017). Moreover, *O*-GlcNAcylation of MAVS by D-glucosamine was demonstrated to be required for influenza virus-induced MAVS K63-linked ubiquitination and the facilitation of subsequent IRF3 activation and IFN-β production (Song et al., 2019).

NF-Kappa-B essential modulator is a crucial regulatory adaptor that is involved in both the IRF-mediated type I-IFN production signaling pathway and NF-κB-mediated proinflammatory pathway. This protein can be ubiquitinated by the E3 ubiquitin ligase TRIM29 at K183 via a K48 linkage, leading to the inhibition of IRF signaling (Xing et al., 2016).

## Role of PTMs in Influenza Virus-Induced Cell Death

To subvert the host response, influenza virus induces early cell death of host cells through the manipulation of PTMs on the viral proteins PB1-F2, M2, and NS1 as well as on the innate immune factor ZBP1. PB1-F2 contains multiple PKCα phosphorylation sites, including S35 and T27. Functional analyses revealed that these two PB1-F2 phosphorylation sites play an important role in influenza virus replication and promote cellular apoptosis in primary human monocytes (Mitzner et al., 2009). M2 interacts closely with HSP40 and p58 (IPK) to form a stable complex, which leads to PKR autophosphorylation and activation, consequently inducing cell death (Guan et al., 2010). Moreover, the H7N9 influenza virus NS1 upregulated p53 expression through enhancing the phosphorylation levels of p53 and facilitating mitochondrial dysfunction, which may initiate NS1-induced apoptosis in human A549 cells (Yan et al., 2016). ZBP1 is an innate sensor of IAV infection (Kuriakose et al., 2016), and its activation contributes to IAV-induced cell death. Mechanistically, during influenza virus infection, RIG-I-MAVS signaling, ZBP1 ubiquitination, and vRNP sensing by ZBP1 together contribute to the activation of ZBP1, resulting in the activation of programmed cell death pathways (Kesavardhana et al., 2017).

## Role of PTMs in Influenza Virus Host Adaptation

During the process of influenza virus host adaptation to different species, the viruses develop different immune evasion strategies in different species. One study found that human TRIM25 bound to all the tested NS1, whereas chicken TRIM25 preferentially bound to avian NS1, and mouse TRIM25 was unable to bind to any of the NS1 (Rajsbaum et al., 2012) (Figure 3B). Accordingly, NS1 in human cells blocks RIG-I signaling by interacting with both human TRIM25 and human Riplet to inhibit RIG-I ubiquitination, whereas NS1 in mouse cells can suppress RIG-I signaling only by binding to mouse Riplet (Rajsbaum et al., 2012) (Figures 3A,B), suggesting that species-specific strategies are used by NS1 to antagonize IFN production.

To replicate efficiently in humans, avian influenza viruses must adapt their viral RNA polymerase (vPol) to human cells. The nuclear proteins ANP32A and ANP32B function as co-factors for vPol activity (Sugiyama et al., 2015). Polymerase subunit PB2 627 is an important viral factor involved in influenza virus host adaption, and the E627K mutation in this subunit is an adaptive marker of avian influenza virus in mammalian hosts. A species-specific difference in ANP32A was reported to contribute to the host restriction of vPol. More specifically, the unique hydrophobic SIM-like sequence in avian ANP32A (avANP32A) enhanced the interaction of avANP32A with both human (PB2 627K) and avian-signature (PB2 627E) influenza virus vPols. However, human ANP32A (huANP32A) lacking the SIM-like sequence interacts weakly with both PB2 627K and PB2 627E vPols (Domingues and Hale, 2017). However, it was proposed that to replicate efficiently in human cells, PB2 627K vPol may increase the utilization efficiency of huANP32A rather than enhancing the binding activity of this protein (Domingues and Hale, 2017). Thus, for PB2 627E vPol to efficiently adapt to human cells, it must either overcome the weak huANP32A interaction or use huANP32A more efficiently.

The PTMs in HIST1H1C regulate the innate immunity as well as influenza virus replication. Specifically, the HIST1H1C phosphorylation mutant (containing T146A) has a weakened interaction with phosphorylated IRF3, which results in reduced IRF3 binding with the IFN-β promoter and decreased IFN-β production. In contrast, HIST1H1C methylation mutants (containing K34A or K187A) have increased IFN-β levels because they promote the binding of phosphorylated IRF3 to the IFN-β promoter (Liu X. K. et al., 2017). PB2 significantly affects HIST1H1C expression and modifications. Specifically, PB2 627K downregulates Sp1, and thereby decreases the binding of Sp1 to the HIST1H1C promoter, resulting in a reduction in HIST1H1C expression. HIST1H1C feedback then further downregulates DNA-PK and EHMT1/2 expression, consequently decreasing the phosphorylation and methylation levels of HIST1H1C (Liu X. et al., 2019). Therefore, PB2 627 regulates influenza virus host restriction via the regulation of HIST1H1C expression and modification.

## Concluding Remarks

As an obligatory intracellular parasite, influenza virus heavily depends on host cellular factors and host cellular machineries to accomplish its life cycle and establish a successful infection. PTMs allow for the dynamic and reversible control of protein functions by modulating the protein abundance, interactors, catalysis, or localization. PTMs also act in many signaling pathways, such as those related to gene regulation, epigenetics, differentiation, protein degradation, tumorigenesis, and immune signaling. Furthermore, PTMs are important parts of the host immune response and play essential roles in replication for many viruses. For influenza virus, PTMs are involved in all steps of the viral replication cycle. The extensive usurpation of various host PTM-mediated pathways by influenza viruses at different stages of their life cycle emphasizes the crucial importance of these cellular machineries in cell physiology and function, and as a consequence, their importance for viral replication and viral diversion of cellular immunity. Currently, manipulation of the host PTMs systems is emerging as a key theme in terms of viral pathogenesis. Accumulating research has found clear evidence of viral proteins that mimic or redirect host PTMs to modify the cellular environment and influence the balance between normal and pathogenic cellular signaling in favor of virus persistence or efficient replication. However, given the wide sequence variation of the known candidates, it is a rather complicated task to systematically identify their viral analogs for ubiquitin or SUMO enzymes. In addition, proteins within released virions also contain multiple types of PTMs, although the function of these modifications within the virions is not currently well understood (Hutchinson et al., 2012).

The complexity of the interplay between viruses and the PTMs systems poses an exciting challenge for future research aiming to unravel the crosstalk between all of these modifications and assess the outcomes at a global level, which may shed further light on the complexity of influenza virus biology. Moreover, questions regarding whether all PTMs impact protein functions during virus infection or lead to dynamic changes in virus infection remain to be answered. There are also likely to be additional, as yet undiscovered, modes of post-translational regulation that occur on influenza virus proteins; their discovery will continue to add to our understanding of the role of PTMs systems during influenza virus infection. Additionally, the mechanisms of how viruses hijack the host PTMs machinery in the process of their coevolution with the host cells remain unknown. Moreover, considering the broad-ranging importance of PTMs in regulating viral replication and the host innate immune response to influenza viruses, more systematic research on this area should bring us closer to controlling and using PTMs for the development of preventative or antiviral therapeutic strategies and may also facilitate the study of innate immunity. Possible directions for future work could include clarifying the functional relevance of all PTMs involved in the specific stages of viral replication and systematically identifying the viral factors that interfere with PTMs.

In addition, given the growing appreciation for various PTMs and their biological functions during microorganism infection, the development of new approaches for studying PTMs is necessary. Presently, mass spectrometry can concurrently identify different types of PTMs, including phosphorylation, acetylation, methylation, and ubiquitination. Future work uncovering the dynamics of PTMs during microorganism infection will provide insight into the regulatory mechanisms and signaling pathways at a system-wide level. Top-down proteomics combined with time-resolved proteomics may reveal coincident modifications on proteins. Moreover, physiological approaches, such as the incorporation of unnatural amino acids with bio-orthogonal reactivity (Neumann-Staubitz and Neumann, 2016), analog-sensitive AS kinases technology (Lopez et al., 2014), mass spectrometry imaging (Buchberger et al., 2018), and spatiotemporal visualization of PTMs using biosensors based on fluorescent proteins and fluorescence resonance energy transfer (Aye-Han et al., 2009; Hertel and Zhang, 2014) should also accelerate our understanding the versatility of PTMs during virus infection.

## Author Contributions

JH and XL drafted and revised the manuscript. LZ contributed to the reference collection and analysis. All authors read and approved the final manuscript.

## Conflict of Interest

The authors declare that the research was conducted in the absence of any commercial or financial relationships that could be construed as a potential conflict of interest.

## Abbreviations

ANP32A: acidic nuclear phosphoprotein 32 family member A
CARD: caspase recruitment domains
CDC25B: cell division cycle 25 B
CDKs: cyclin dependent kinases
CK1 α: casein kinase 1
CNOT4: Ccr4-not transcription complex subunit 4
CRM1: chromosome region maintenance 1 protein homolog
DNMT: DNA methyltransferase
DNMT3B: DNA methyltransferase
GCN5: general control of amino-acid synthesis 5-like 2
HA: hemagglutinin
HDAC1: histone deacetylase 1
HDAC6: histone deacetylase 6
HDAC6/8: histone deacetylases6/8
HIV: human immunodefciency virus type-1
HSP90: heat shock protein 90
IAV: influenza A viruses
IFITM3: IFN-induced transmembrane protein 3
IFITM3: interferon-induced transmembrane protein 3
IFN: interferon
IFNAR1: type I IFN receptor 1
IRF3: interferon regulatory factor 3
M: matrix protein
MAVS: mitochondrial antiviral-signaling protein
MLC: myosin light chain
NA: neuraminidase
NEMO: NF-Kappa-B essential modulator
NES2: nuclear export signal 2
NP: nucleoprotein
NS: non-structural protein
OAS: oligo (A) synthetase
PA: polymerase acidic protein
PB1: polymerase basic protein 1
PB2: polymerase basic protein 2
PCAF: P300/CBP-associated factor
PKC α: protein kinase C α
PKM: pyruvate kinase M
PKR: protein kinase R
PTMs: post-translational modifications
RanBP3: ran-binding protein 3
RdRP: RNA-dependent RNA polymerase RdRp complex
RIG-I: retinoic acid-induced gene 1 protein
SIM-like: SUMO interaction motif-like
TRAF3: TNF receptor-associated factor 3
TRIM: tripartite motif
UIM: ubiquitin-interaction motifs
USP11: ubiquitin–proteasome system protein 11
vRNPs: viral ribonucleoprotein complex
VSV: vesicular stomatitis virus
ZBP1: Z-DNA -binding protein 1.
